# The Old Year

**Published:** 1899-01-07

**Authors:** 


### Hospital Clinics and Medical Progress.

### THE OLD YEAR.

Standing as we are at the commencement of a new year, it is as natural as, indeed, it is commonplace, to run over in one's mind the main features of the year which has gone, and to speculate as to the events which are likely to make memorable the one which is now just opening upon us. So far as regards the position of the medical profession in the public eye, the past year has been marked by striking contrasts, for while many things have happened which have not only redounded to our honour, but have shown in marked degree the position of influence which is occupied by the leaders of our profession, others have occurred which have sent a thrill of shame through the body corporate and have made us feel acutely how by the sins of a few individuals the status of the whole profession may be dragged down.

No one can have passed through the streets of London during recent months without observing how largely the backslidings of medical men have figured among the tit-bits offered by observant editors for the delectation of their readers. Such phrases as "West-End Medical Scandal" or "Doctor in the Dock" or "Medical Man Charged with Manslaughter" have become stock headlines with the evening press, and are flaunted in our face in enormous type at every corner and almost every week. Happily, in many cases it has turned out that the persons charged with these offences have not been medical men at all, while in others the charges levelled at them have been shown to be false; but, unfortunately, a residuum has remained.

### Sins against Law and Morality.

One of the most marked features of the year which has gone by has been the publicity given to the fact that a certain form of crime, to the existence of which some good people have blindly shut their eyes, is rampant throughout the country, and that in some cases medical men have allowed themselves to become the instruments of its commission. The part played by some few misguided medical men in this matter has without doubt led to much blame being unjustly cast upon the profession as a whole. Most unjustly and most unfairly, for the main protest against the prevalence of this class of crime has always come from the medical profession itself.

The events of the past year have made it abundantly clear that, for a long time back, criminal abortion has been widely practised in our midst; that the would-be perpetrators of the crime have been allowed to advertise their proceedings with impunity; and that the public and even the police have looked on with complete indifference so long as death has not resulted from its clumsy performance; and, as we say, while these things have been going on, it has been almost exclusively from the medical profession and from the medical press that protests have arisen as to the laxity of the public conscience in this matter. The exposures of the past year in regard to the prevalence of this crime have involved a terrible stirring up of social mud, and if a little of it has stuck here and there upon a very small number out of the 35,000 medical practitioners whose names appear on the Register, we must not make too much of it. It is a stain, no doubt, and much to be regretted; but, if we consider how few have given way to these women who beg and pray to be delivered from their trouble, and who, as is made manifest by the cases that have come to light, are ready to brave anything, even serious risk of death, if they can but be "put right," we seem to see—what, indeed, is the truth—that the exceeding prosperity of the advertising quacks, with their sham remedies for "irregularities," is but a testimonial to the virtue of the profession, and that medical men are made of sterner stuff than some imagine.

### Honours to the Profession.

But let us turn to pleasanter themes. Honour has been done to members of the profession in many ways, by titles, by decorations, and by promotion in the services. One medical officer, Surgeon William Job Maillard, M.D.Lond., of the Royal Navy, has obtained the Victoria Cross, which was conferred upon him for conspicuous bravery during the outbreak at Candia. Then, among the honours conferred on the profession, we must refer to the Royal warrant constituting the Royal Army Medical Staff Corps which was issued in July last. No doubt something still remains to be done in various directions to place the army medical service on a perfectly satisfactory footing, but the great thing has been done; it is no longer possible to regard the medical officers as mere civilians; they form a Royal corps; they have control over their men; they have the same titles as officers in other branches of the service, titles recognised throughout the army as indicating rank. This is but tardy justice; but that the position so long demanded should at last be granted, and granted in handsome terms, is a proof of confidence, a sign of recognition of professional position, and thus must be classed among the honours done to the profession in the year 1898.

### The General Medical Council.

The General Medical Council has done a great deal of work, but has not a very great deal to show for it. Much of its time has, indeed, been occupied in putting its house in order, in questions as to procedure, as to the rights of its members, and other matters which, however essential they may be to the doing of its work, do not themselves represent work done. Then, again, a good deal of time is of necessity taken up in the hearing of penal cases—that cleansing of the Register which, we suppose, is one of the first duties of the Council.

Of work which has an external bearing, perhaps the three most important acts of the Council have had to do with—(*a*) raising the standard of the preliminary examinations by the deletion of some which were not considered satisfactory; (*b*) the insistence upon a statutory declaration as to the facts of any case before it is brought before the committee for the consideration of penal cases; and (*c*) the laying down of certain principles which are regarded as essential in any measure for the control of midwives. Among these principles is the very important one that not only should the licensed midwives be properly educated, but that unlicensed women practising midwifery, whether calling themselves midwives or not, should be liable to a substantial penalty. That the Council should insist upon this as an essential condition is a matter of great moment, for such a provision would make the control of midwives a reality.

### London Water Supply.

The past year has been characterised by a renewed interest in all that concerns the water supply of the metropolis. The occurrence of another water famine in the East End has, above all things, emphasised the necessity of having proper intercommunication between the pipes of the different companies, so that in future it shall be impossible for any defaulting company to plead want of water as an excuse for giving short supply at a time when there is plenty to be had in the areas supplied by other companies. This is a simple and common-sense precaution, which ought to have been put in operation long ago; and it is one, moreover, which, besides directly preventing the recurrence of water famines, is likely to have a very salutary indirect effect upon the management of the water companies themselves. It seems to be admitted on all hands that much of the scarcity in the East of London was due to leakage and to waste, and it is not unnaturally asked, why is this leakage and waste allowed to continue? It is not difficult to understand that so long as water is plentiful it is cheaper to pump it into the mains than it is to keep a constant eye upon all possible sources of waste, and that if the companies are allowed to cut short the supply whenever their reservoirs run empty there is but little temptation to adopt stringent measures against leakage. Intercommunication of pipes, however, puts the matter on a very different footing. Any company that has not enough water will in future have to buy from its neighbours, and this is just what the companies will not like to do. Thus the past year has seen at least one step forwards in regard to this weary matter of the London water supply.

### Typhoid Fever and Impurity of Soil.

The very important discussion which took place at the Royal Medical and Chirurgical Society, at the beginning of the year, on the prevention of enteric fever, was marked by an announcement by Sir Richard Thorne of the results of some observations which were being made by Dr. Sidney Martin for the Medical Department of the Local Government Board in regard to the real relation between soil impurity and that disease. These observations were being undertaken with the object of ascertaining how it was that enteric fever clung to certain towns and places, whilst it did not do so elsewhere; and he found that, by taking certain soils containing a certain amount of organic matter, that is to say, organically polluted soils, and then sterilising them, the enteric fever organism, when introduced into the soil, had lived for a long period—namely, for 268 days (since then this period has been extended to 456 days; see Report of Medical Officer to Local Government Board, 1897-98); whereas in soil containing no organic matter, but similarly sterilised, it could only live for from 14 to 25 days. This gives the keynote to the modern view as to the influence of the soil in maintaining the endemic character of typhoid fever. In the article on "Water-borne Diseases," in the "Twentieth Century Practice of Medicine," published last year, the following occurs: "It is not a mere question of washing accumulations of filth off the surface, but of draining out of the soil itself organisms which have grown in it—the descendants doubtless of microbes which have been implanted in it by previous contamination by typhoid excreta, but themselves quite innocent of any direct connection with any recent case of typhoid fever." If to this we add what Dr. Martin and others have shown, that this persistent presence of the typhoid bacillus in the soil depends on the fact that the soil itself is foul, we are able to explain much which is of importance in regard to the etiology of stray cases of typhoid fever.

### Plague.

Plague has remained a constant matter of interest throughout the year. Not that it has greatly enlarged the area in which it has been prevalent, but it has again and again broken bounds, and rumours of its occurrence in fresh places have constantly been current. On four occasions infected ships have reached our shores, but fortunately, so far, no damage has been done. Rumours of the extension of the disease have also come from the east coast of Africa, and it has attacked certain districts in Eastern Russia with great virulence. In relation to the possibility of its extension westwards it may be noted that, great as has been the destruction wrought by the plague in Bombay and other towns of India, its proportional mortality has not approached what is recorded in history about the outbreaks in times gone by. Still, it is a matter about which it is impossible to avoid a certain degree of anxiety. The unknown is always alarming, and it has to be confessed that, however much we may hope and may believe that the infection of plague would fizzle out if it were implanted in such a country as England, we do not know. During the year vaccination against the plague, as performed by M. Haffkine, has had a great vogue, but how far the security given by it is to be relied upon is still a question. Perhaps the most striking bit of knowledge that the year has produced in regard to plague is the observation of Dr. Simond as to the part played by rats in the spread of disease, and especially his assertion that the actual implantation of the infective material is due to the fleas and other parasites which abound both on the bodies of the affected rats and in the dwellings where plague becomes, what it has long been recognised to be, a "house disease.".

### A Crusade Against Consumption.

The institution of an organised attempt to put some limit to the ravages of consumption and other forms of tuberculosis is no doubt one of the most striking events of the year. It has been by no means creditable to the medical profession, and certainly has spoken ill either for its own faith in the dogmas of its scientific leaders, or for its influence with the general public, that such indifference should continue to be shown to the ravages of a disease which is held to be preventable. The truth probably is that the profession at large has not really and in its heart believed all that it has been told as to the curability and preventability of consumption.

Every practitioner has seen a case here and there which has got well, and a good many more which have appeared likely to do so but have just missed their goal, but the vast majority of the cases have died, and what has been drummed into our ears about the curability of phthisis has, to the mass of the profession, been to a large extent a matter of book knowledge, or has had to do with the comparatively small number who could go sea voyages or visit the high Alps. So far as consumption has affected their poorer patients, that is the mass of their patients, consumption has remained, and still remains in the eyes of most practitioners, a desperately, an almost hopelessly, fatal disease. Hence every effort to control it has been hampered by want of faith and by a paralysing hopelessness as to the utility of individual effort. This year, however, has seen a great revival of endeavour to stop the progress of this curse, which has for so many centuries dogged the steps of civilisation, and the efforts are no longer to be individual, but national and concerted. Let us hope they will succeed.

### The University of London.

One of the most important events of the year, so far as regards medical education in London, has been the passing of the Bill appointing a Statutory Commission to undertake the reconstitution of the University of London. At last there is a possibility that dwellers in the metropolis may see London in possession of a real teaching university which shall give to its students the advantages of a university training in a far larger degree than has been possible under the arrangements which have hitherto existed.

### Vaccination.

One of the most important events of the year has been the passing of the Vaccination Act, by which the whole of the public vaccination machinery has been entirely altered. Ever since the days of Jenner-arm-to arm vaccination has been looked upon as being, what indeed it still is, the most certain and successful mode of producting immunity to small-pox, and although the Government has distributed vaccine lymph, preserved in various ways, this has always been done with the direct object of starting a series of arm-to-arm vaccinations to be kept up by the vaccinator as long as possible. Now, so far as public vaccination is concerned, this is abolished, and recourse is to be had to glycerinated calf lymph or such other lymph as may be issued by the Local Government Board. The advantages will be great, the disadvantages will not be small, and among the latter must be placed the divided responsibility as to the character of the lymph. Under the old arrangement, a clever and careful vaccinator could keep up a good strain of lymph by which he could easily produce admirable results—and his care was rewarded. Under the new arrangements, a careless man has but to throw the blame of ill-success upon the lymph he gets from head-quarters, and no one can prove that he is not right. There is certainly not the reward for good work that there used to be.

### Law and Conscience.

The passing of the Vaccination Act was marked by humiliating concessions to faddists which will, we fear, be quickly taken advantage of by the opponents of law and order. Never was there a better example of the danger which always arises from adopting the fatal plan of giving way for the sake of peace—of trying to reconcile the irreconcilable. Parliament chose to shut its eyes to perfectly well-known facts, and to act as if conscience was the difficulty with the anti-vaccinists. It was even said that martyrs make proselytes, and that vaccination would be more universal if compulsion were not enforced. This was a perfectly blind and unwarrantable assumption, contrary not only to all knowledge of human nature, but to all the experience which had been gained during the years of waiting for the Report of the Royal Commission, a period of practical non-compulsion. The conscience clause was passed purely for the sake of political peace; it was a mere sop to Cerberus, a sop which Cerberus has ragged and shaken and made ridiculous, but has refused to swallow or be satisfied with, and now we have to face the consequences. Among these are a widespread contempt for an Act of Parliament—a contempt which unfortunately is not confined to common people, but is shared in and expressed by magistrates and people of influence and education; a determination on the part of many to put the law at defiance; and an expressed intention to urge local bodies to flout the national will as expressed by the national Parliament. To those who recognised, as all ought to have done, that to the anti-vaccinist "conscience" was but a sham and a stalking-horse, it comes as no surprise to find that no sooner has the conscience clause been obtained than it is thrown aside as an ignoble means of defence, unworthy of those whose true aim is to maintain the right of man to do as he likes, independently of the government under which he lives and of the social system of which he forms a part. When we find that people are now being urged to have nothing to do with the conscience clause, and that guardians are being urged to maintain their attitude of defiance of the central board, and apparently are not unwilling to accept the suggestion, we cannot shut our eyes to the fact that far greater issues than vaccination are at stake, and that what the Government will next have to decide is whether law is to prevail or not. It would have been easier to have grasped the nettle at first while it was but a tender plant. Then it was a question of vaccination alone, about which the majority were as indifferent as they were ignorant. Soon it will be a question of "anti" everything, an issue most difficult to control. Yet how ridiculous the whole thing seems. If vaccination is no use, let us say so. If its efficacy is doubtful, let us abolish compulsion. If it is not doubtful, let us enforce it. But to maintain compulsion and yet put in a conscience clause—and such a conscience clause—is beneath contempt. Fortunately, it is only for five years.

